# Quality of Care of Young People Presenting With Self-Harm at the Norfolk and Norwich University Hospital Emergency Department

**DOI:** 10.1192/bjo.2025.10637

**Published:** 2025-06-20

**Authors:** Bolanle Mojibola, Ayomipo Amiola, Tosin Daropale

**Affiliations:** Norfolk and Suffolk Foundation Trust, Norwich, United Kingdom

## Abstract

**Aims:** To determine the quality of care received by young people (16–25 years) presenting with self-harm at the Norfolk and Norwich University Hospital Emergency department using NICE guidelines QS34.

**Methods:** Retrospective data collection from electronic patient records.

These patients referred via the emergency department of the Norfolk and Norwich University Hospital were assessed as a one off.

271 patients presented with self-harm behaviour to the Accident and Emergency of the Norfolk and Norwich University Hospital in July and August 2024. They were all referred for assessment to the Mental Health Liaison Service. 85 of these patients were of ages 16–25. All the 85 were audited.

**Results:** 84.7% of patients had a record of risk assessment to reflect if there were any immediate concerns about their safety while 11.8% did not have a record of risk assessment. 3% left before assessment.

81.2% had mental state examination done, 15.3% did not have a mental state examination. 3% left before assessment.

78.8% had an initial assessment of safeguarding concerns, 17.6% did not, and 3% left before the assessment.

82.3% had a collaboratively developed care plan, 11.8% did not and 5% of them either left before the assessment or did not engage with care planning.

80% had initial assessment of social circumstances, 12% did not and 5% either did not engage or left before the assessment.

More than 80% compliance was achieved within the areas of assessment except for safeguarding concerns which was only 78.8%.

**Conclusion:** Positively, the trust has a template which is aligned with NICE standards. If followed, the guidelines will be adhered to. Assessments that did not meet the guidelines did not use the template. Other NHS trusts should ensure their Electronic patient records have the same and provide regular training to staff.

It is useful to familiarize new/locum staff about the importance of the different aspects of assessment and to follow the guide of the template provided on EPR.

The content of these assessments varied, some had comprehensive assessment while others were less detailed. This should be investigated in the future to determine the definition of an adequate assessment across board.

